# Arctic Climate Change, Economy and Society (ACCESS): Integrated perspectives

**DOI:** 10.1007/s13280-017-0953-3

**Published:** 2017-10-24

**Authors:** Anne-Sophie Crépin, Michael Karcher, Jean-Claude Gascard

**Affiliations:** 1 The Beijer Institute of Ecological Economics, The Royal Swedish Academy of Sciences, Lilla Frescativägen 4, Box 50005, 104 05 Stockholm, Sweden; 2 The Stockholm Resilience Centre, Stockholm University, Kräftriket 2 B, 10691 Stockholm, Sweden; 3 Ocean Atmosphere Systems GmbH, Tewessteg 4, 20249 Hamburg, Germany; 4 Alfred-Wegener-Institut Helmholtz-Zentrum für Polar- und Meeresforschung, Bussestrasse 24, 27570 Bremerhaven, Germany; 5 LOCEAN, University Pierre and Marie Curie, 4 Place Jussieu, 75005 Paris, France

**Keywords:** Arctic Ocean, Climate change impacts, Economic activities, Environmental protection, Sea ice, Society and governance

## Abstract

This introduction to the special issue presents an overview of the wide range of results produced during the European Union project Arctic Climate Change, Economy and Society (ACCESS). This project assessed the main impacts of climate change on Arctic Ocean’s geophysical variables and how these impending changes could be expected to impact directly and indirectly on socio-economic activities like transportation, marine sea food production and resource exploitation. Related governance issues were examined. These results were used to develop several management tools that can live on beyond ACCESS. In this article, we synthesize most of the project results in the form of tentative responses to questions raised during the project. By doing so, we put the findings of the project in a broader perspective and introduce the contributions made in the different articles published in this special issue.

**Electronic supplementary material:**

The online version of this article (doi:10.1007/s13280-017-0953-3) contains supplementary material, which is available to authorized users.

## Introduction

During the twentieth century, human activities have become globalized. While these developments have led to amazing improvements in human wellbeing, they have also resulted in global environmental problems, like climate change, which now challenge the future wellbeing of the human population on Earth (Steffen et al. 2015).

Specific impacts of climate change are exceptionally dramatic in the Arctic, with greater temperature increases compared to the Earth as a whole, due to polar amplification (IPCC 2013). Climate change is expected to transform the Arctic Ocean from a year round frozen sea with multiyear ice to a sea with open waters in summer and annual ice in the winter similar to the Antarctic Ocean. Such dramatic change will likely have sizeable impacts on marine ecosystems, economic activities and indigenous and local peoples in the Arctic. The Arctic Ocean provides essential global climate regulation and substantial ecosystem services and benefits to humanity also outside of the region—all of these aspects may be affected. The Arctic environment, human society, and its economic activities are connected to each other, forming a complex adaptive system (Norberg and Cummings 2008). Climate change impacts are likely to hit multiple parts of the system either simultaneously or separately with delays, making it particularly challenging to assess overall impacts and how to deal with them (Arctic Council 2016; Crépin et al. 2017). Climate change is likely to reinforce socio-economic activities in the region making it all the more a true social–ecological system (Berkes and Folke 1998). Interactions between the different parts of the system occur across spatial and temporal scales and Arctic resources are becoming a global concern when resource stocks in the rest of the world deteriorate, while population is growing.

This *Ambio* special issue highlights some of the scientific results produced within the European Union (EU) project Arctic Climate Change Economy and Society (ACCESS) 2011–2015.

ACCESS had multiple objectives:

- Continue to monitor and analyze changes in the Arctic Ocean following work in previous EU projects (DAMOCLES^1^ and ATP^2^), with the aim to provide accurate estimates of current status, changes, predictions and uncertainty estimates of future developments up to 2040, improve observation infrastructure, and assess forecasting capabilities in particular regarding sea ice and weather in the Arctic.
- Analyze the direct and indirect impacts of climate change on principal economic activities like shipping, tourism, fisheries, aquaculture and resource extraction.
- Analyze cross-sectoral issues like impacts on Arctic marine ecosystems, the need for marine protected areas, challenges of providing essential Arctic infrastructures and effects on local and indigenous peoples.
- Give an overview of the regulatory systems, legislation and agreements governing relevant Arctic economic activities and assess strengths and weaknesses of the governance system, its response to natural and human generated stress and its relevance to sustainable development in the region.
- Improve integrated management capacity for the Arctic with appropriate management tools.

In this article, we provide an overview on some of the contributions from ACCESS to those objectives with an emphasis on the cross-sectoral perspective. A full list of ACCESS publication as of autumn 2017 can be found in Supplementary material S1. The project provided contributions related to the impacts of climate change on the natural environment in the Arctic. Some examples include new direct and satellite-based observations of sea-ice properties (Hwang et al. 2015; Divine et al. 2016), atmospheric conditions (Gascard et al. 2017 and references therein) and sea state (Dmitrenko et al. 2014; Oziel et al. 2016); development of a method to design better observation networks (Kaminski et al. 2015); data analysis and modelling to improve scenarios of future sea ice and ocean (Gascard 2012; Gascard et al. 2017). The project used much of these findings to inform studies on the impacts of climate change on society including costs and benefits of off-shore resource extraction (Petrick et al. 2017), tourism, marine transportation, salmon (*Salmo salar*) farming in Norway, and different scenarios (model or narrative based) of future development for example for the maritime Arctic (Brigham 2015) and fisheries (Eide 2017). The project identified and quantified environmental impacts of emissions from different sources and activities in particular regarding oil spills and response capacity (Nordam et al. 2017; Wilkinson et al. 2017); air pollution from ships (Law et al. 2017; Schröder et al. 2017); and noise. Several syntheses were produced for example on food chain interactions in the marine Arctic (Crépin et al. 2017), seafood production (Troell et al. 2017) and existing governance regimes and gaps (NERC 2015). Finally, the project contributed tools for research and management support like a data management system (Godøy and Saadatnejad 2017), an advanced Arctic Ocean observing system (IAOOS) (Gascard 2012), a pan-Arctic marine spatial planning tool (Edwards and Evans 2017), a framework for integrated ecosystem-based management (Crépin et al. 2017) and a set of Arctic indicators for sustainable development (NERC 2015). The project also organized two workshops dedicated to indigenous peoples and two transdisciplinary PhD courses, published newsletters, and policy briefs.^3^ In the following, we will illustrate the role of most of these contributions in a broader context, as answers to eight questions related to Arctic development under climate change.

## Eight questions related to Arctic development under climate change

### I. How do we expect sea ice to change in the Arctic over the next three decades?

Satellite-based observations have documented a drastic reduction of the Arctic sea-ice area and extent over the past 30 years in all seasons, with a higher rate of decrease in the first decade of this century (Stroeve et al. 2012). In addition, mean sea-ice thickness declined from more than 3 m down to less than 2 m (Renner et al. 2014; Lindsay and Schweiger 2015), leading to a stark reduction in multiyear ice (Comiso 2012). Other Arctic sea-ice characteristics have also started to change and will likely continue to do so, like the length of the sea-ice-free season, earlier break up and later freeze up, the occurrence of melt ponds and the under ice topography (e.g., Hwang et al. 2015; Divine et al. 2016). All of these changes must be documented and understood, as they all play a role in the Arctic climate system, but they also have a direct impact on the human use of the Arctic.

Earth system models are widely used to project the development of climate and its components (also Arctic sea ice area and thickness) for future decades. These models are driven by natural forces, like solar radiation, and their climate simulation is modified by anthropogenic influence, e.g., due to the release of greenhouse gases, which act as so-called climate forcers (IPCC 2013). When developing an understanding of possible futures of the Arctic climate system, it is important to consider the different uncertainties contributing to the total uncertainty for a specific forecast time period (i.e., in our case three decades). Uncertainties come principally from model errors, i.e., misrepresentations of natural processes in numerical models, uncertainties about the actual development of future greenhouse gas emissions and natural variability. The uncertainties due to model differences and natural variability play the largest role within the forecast period of 30 years that we envision. In particular, very long-term natural variability, like the Atlantic multi-decadal oscillation, seriously hampers the potential accuracy of a forecast for timescales of several decades. Climate forecasts in the order of 30 years thus have large uncertainties due to large natural variability, in particular at high latitudes, most likely due to climate feedbacks in these areas and the difficulties of their representation in models (see, e.g., Hawkins and Sutton 2009). So due to the nature of the Arctic climate system, multi-decadal predictions have their limits, as have seasonal predictions (Serreze and Stroeve 2015).

The prospect of a continuing decline in Arctic sea-ice-cover, in particular in the southernmost areas, makes an increase of human activities in the Arctic, such as shipping, tourism, resource extraction and fisheries, extremely likely. Thus in addition to interest driven research for understanding the dynamics of the Arctic climate system, society has a growing need for predictions about the Arctic environment, in particular for sea ice, as knowledge about its abundance and state (thickness, concentration) is of great relevance for all human activities beyond the shorelines, now and in the future. Any attempt to project possible future development of economic sectors in the Arctic must account for the uncertainties described above. Excluding models that did not produce results comparable to observations during the observations period could possibly reduce one source of uncertainty (Massonnet et al. 2012; Wang and Overland 2012; Snape and Forster 2014).

In ACCESS, we have contributed to these needs with numerous direct and satellite-based observations of sea-ice properties (see references in Gascard et al. 2017), ocean observations (Oziel et al. 2016) and by using numerical models to evaluate the possible development of sea ice and ocean over the coming three decades (see references in Gascard et al. 2017). This information was also used to evaluate possible developments in economic sectors in the Arctic in the future (see Crépin et al. 2017; Eide 2017; Petrick et al. 2017; Troell et al. 2017).

Such an approach was used in the ACCESS project to evaluate possible developments in economic sectors in the Arctic in the future (Gascard et al. 2017). For example, the skill in reproducing the observed seasonal cycle of the sea-ice extent in relevant areas for the respective economic activities was used as an indicator (e.g., Petrick et al. 2017). Despite a general trend of further reduction of summer sea ice and increase in the length of the sea-ice-free season in the Arctic Ocean margins, these model simulations suggest obstacles for free shipping (Gascard et al. 2017); even in future periods of very low sea-ice-cover passages could be blocked. This, and the fact that human activities would not be restricted to the summer season, explains the necessity for seasonal to short-term sea-ice forecasts, for planning and navigation purposes. However, improving the quality of Arctic weather forecasts will be a key requirement, for reliable sea-ice prediction on a weekly timescale, as well as the forecasts of air temperature, icing and wind conditions, which are important for safety. These have currently too low accuracy. Better Arctic weather forecasting and suggestions for how to improve it, taking into account the requirements of a future observing network, were identified (Anderson and Sato 2012). Efforts to enhance the extraction of information from polar orbiting satellites along with an increase of direct atmospheric observations in the interior Arctic are essential elements. This could include surface buoys such as IAOOS^4^ platforms equipped with atmospheric, ice and ocean sensors and an increased density and/or frequency of radiosonde releases to be used to improve Arctic weather forecasts.

Thus despite the general tendency of reduced sea-ice-cover over the coming decades, a wide range of possible developments will still need to be taken into account for planning human activities over this time range. Further research, improved observations, and further improvement of methods to forecast will be essential to provide the baseline for decision making.

### II. What are the expected impacts of climate change on live marine Arctic resources?^5^

There is substantial evidence that climate change is having an impact at multiple levels of the Arctic food chain from several species of ice algae all the way up to the top predator Atlantic cod (*Gadus morhua*). Warmer water is likely to change the availability of melting floating ice, which could influence primary producers’ composition in the Arctic waters, in particular ice algae. These changes could trigger a decrease in *Calanus glacialis*, the dominant (80%) zooplankton species to the advantage of *C*. *finmarchicus* (Ellingsen et al. 2008). *C. glacialis* is a much more lipid-rich species and this change would have direct negative impacts on higher trophic levels (Falk-Petersen et al. 2009) like herrings (*Clupea harengus*), which in turn are essential prey fish for higher trophic levels. In contrast, inflow of warm water to the Barents Sea favours herring recruitment (Sætre et al. 2002). Changes in currents and water masses could also influence capelin (*Mallotus villosus*) distribution and migration (Bogstad et al. 2000). Collapses in the capelin stock may result from increased food competition and herring predation on capelin larvae (Gjøsæter et al. 2015). Sea temperature and other oceanographic changes are also likely to directly affect recruitment and growth of Atlantic cod, the main predator of capelin (Ottersen et al. 1998).

Indirect effects of climate change are less studied but changes in trophic levels could influence prey patterns, competition between species, and parasitism. Marine invasive species, like crabs, could expand to sub-Arctic and Arctic waters even under moderate climate change scenarios (De Rivera et al. 2007), due to warmer waters and increased human activities. In particular, the red king crab (*Paralithodes camtschaticus*) benefits from warmer ocean temperatures and already supports valuable fisheries (Hjelstedt 2012) but may also lead to predation on native species and habitat destruction (Falk-Petersen et al. 2011). Ocean acidification and increased CO_2_ emissions could inhibit growth of shells leading to crab mortality (Long et al. 2013).

Each of these studies addresses some partial aspects of the impacts of climate change. An important contribution of the ACCESS project is to provide a description of the impacts of climate change at system level, based on literature studies and theories (Crépin et al. 2017), modelling (Eide 2017) and observations (Oziel et al. 2017). The integrated picture presented in Crépin et al. (2017) builds on most ACCESS work. It reveals for example that the increased fluctuations in stock biomass and stock age composition are likely to remain limited compared with normal environmental fluctuations in this area and market fluctuations (Eide 2017). Ocean acidification could potentially impact many parts of the ecosystems and the economic activities tied to them (Crépin et al. 2017). New economic activities developing in the Arctic Ocean are also likely to influence Arctic marine resources, as investigated in ACCESS, through increased pressure on the environment due to increased pollution from oil spills (Nordam et al. 2017; Wilkinson et al. 2017) and maybe air pollution (Law et al. 2017). Market changes could substantially influence the demand for these resources (Crépin et al. 2017; Petrick et al. 2017; Troell et al. 2017) and economic activities could compete in using sensitive ecosystem areas (Edwards and Evans 2017).

### III. How does climate change influence the provision of ecosystem services supporting fisheries and aquaculture?

The complex interactions between different impacts of climate change and the lack of observation data make it challenging to clearly predict the implications on the provision of goods and ecosystem services from Arctic seas (see, e.g., Post et al. 2009). Results from the ACCESS project reported below provide a more complete picture.

Model predictions (Eide 2017) indicate a 10% increase in carrying capacity and a larger distributional area in the Barents Sea for demersal species (with seasonal variation) but the key fishing areas remain, allowing “business as usual”.

While aquaculture is currently limited mainly to Norwegian Atlantic salmon farming, temperature increase will open new areas for farming primarily in north Norway and the Kola Peninsula. Increased productivity is likely to make the industry more attractive. Other impacts of climate change may also affect pathogen distribution and incidences, frequency of storms and thus create damage to the farms and freshwater runoff. Conservation and tourism interest could also compete with the farming activities (Troell et al. 2017).

Indirect impacts of climate change are likely to influence the provision of goods and services from Arctic marine ecosystems at least as much as direct impacts, through changes in input and product markets and other socio-economic factors. Climate change may impact fisheries input markets, for example via changes in fuel pricing (carbon pricing). Today, fuel is often subsidized but there is demand for a global carbon price although such policy presents many challenges, including difficulties to reach agreements and carbon leakage^6^ when the price differs among countries. Environmental concerns could also put demand pressure for certified fisheries, and eco-labelled products. Eco-labelling today focuses on ecological and management issues rather than carbon footprints (Troell et al. 2017).

The fishing nations in the Arctic Ocean—in particular Norway, Russia, Iceland and the EU—seem to have different understandings of the sustainability concept, which sometimes leads to disagreement regarding how to handle bycatches and the size and distribution of fishing quotas. Climate change is likely to exacerbate such disagreement by triggering new movements of fish stocks across national borders (Stammler-Gossman 2015; Oziel et al. 2017).

In addition, fishing is perceived as a fundamental right of Coastal Sami in Norway. The Finnmark (Norway) and Murmansk (Russia) regions strongly depend on each other. These regions cooperate around fish landing, services and labour. The Norwegian fish processing section lacks labour force, while fish shortage is a main issue in Murmansk. Different people perceive the climate induced changes very differently depending on their initial knowledge and cultural background. Fishermen in different countries also have different catch strategies, either waiting for fish stocks to move to convenient fishing places, or following the fish using large ships (Stammler-Gossman 2014). Resource users seem to manage their resource better if they are aware that poor management could seriously harm the resource (Lindahl et al. 2016).

### IV. What economic activities are likely to expand in the Arctic due to climate change?

In addition to the likely increase of seafood production activities, climate change could influence non-renewable resource extraction, marine transportation, and tourism. A significant share of the world’s undiscovered oil (13%) and natural gas resources (30%) are assumed to lie under the seabed of the Arctic Ocean (USGS 2008a, b; Gautier et al. 2009). Gradual warming has improved the accessibility of the Arctic Ocean and raised hopes among hydrocarbon producers who envisage to diversify their portfolios away from less politically stable or depleting sources elsewhere. Oil and gas importers in Europe and Asia also wish for reduced dependence on traditional suppliers in Russia, the Middle East, or Africa that are perceived as geopolitically risky. The fluctuations of energy prices will be crucial for energy developments (Emmerson and Lahn 2012) as well as the level of international cooperation and climate policy (Overland et al. 2015). There is evidence that the Arctic’s relevance for international gas markets will likely decline and for oil markets at least not increase (Lindholt and Glomsrod 2012).

ACCESS research specifies and quantifies the substantial cost of bringing Arctic resources to markets (Petrick et al. 2017). The need for special, winterized equipment, including ships and platforms and long distance from support infrastructure make exploration and production activities in the Arctic Ocean especially costly compared to other, even non-conventional sources of hydrocarbons. The challenges posed by temporary sea-ice-coverage, harsh weather conditions, darkness, remoteness of the fields, and lack of infrastructure such as search and rescue (SAR) facilities have up to now hindered exploitation of these resources offshore. ACCESS results show that projects will require a high market price and more cost-effective technology to attract investments. Simultaneously, the pristine Arctic ecosystems are seen as being in danger of pollution by oil and gas production facilities in shore, transportation, and associated infrastructure (Dalsøren et al. 2013; Petrick et al. 2017). For example, the Yamal Peninsula gas field is currently a fully active enterprise.

Despite the most recent price drops (since 2014 oil price is around 50 USD per barrel in contrast to around 100 USD earlier), Arctic oil and gas production cost estimates are still just below the current world market price for oil and the average European gas price. Additional costs for local infrastructure provision in the widely undeveloped Arctic are location-dependent, highly uncertain and likely not fully taken into account. The reduction of sea ice might facilitate access to the Arctic Ocean, but could also impact wave conditions in the once ice-covered areas, which may increase the cost of transportation and off-shore production. Scenario results, developed during the ACCESS project, suggest that in 2040 the ice will have receded enough to make gas production technologically feasible in the European off-shore Arctic under most emission scenarios (Petrick et al. 2017). However, recent oil and gas price developments, which give some indication of the upper limit of the highest marginal production cost in the market today, suggest that Arctic offshore oil and gas will not be competitive in the near future. Under these circumstances, the large estimated offshore oil and gas resources will likely remain untapped as long as purely economic reasons determine the development decision (Petrick et al. 2017).

The decrease in sea-ice extent has improved the feasibility of seasonal Arctic routes for commercial shipping activities between Asia and Europe, which has led some to predict significant increases in shipping volumes through the Arctic.^7^ The interest in Arctic trans-shipping routes^8^ stems from the fact that these routes offer shorter transit between Asia and Europe for some port pairs compared to alternatives (via the Suez Canal), and thus potentially reduce journey times and travel cost.

Among many other aspects, ACCESS research on marine transportation focused on a scenario narrative developed during the project to illustrate possible maritime developments in 2040 (Brigham 2015) and different aspects affecting transport costs (Morgenroth 2014). In 2014, exports between European and Asian trading partners excluding Taiwan, who would benefit from a shorter route through the Arctic was worth 772 thousand million USD, or roughly 4.2% of world exports.^9^ If the Northern Sea Route (NSR) was like the Suez route, then there would be significant shift of shipping activities. However, this depends on the profitability of the NSR compared to the Suez route and its feasibility in terms of support infrastructure, SAR capabilities and meteorological and oceanographic support, which cannot be taken for granted (Morgenroth 2014). The profitability of the NSR depends on the routes’ relative shipping costs. The distance at sea that needs to be covered has a significant bearing on shipping costs, but those also depend on the sea conditions, as fuel costs are considerably higher if ice is present, and other costs incurred such as ice-breaker costs, canal fees and insurance costs.

The potential development of the NSR will depend on the development of a modern infrastructure. Significant shipping activities without a more developed infrastructure are risky for the vessels. Currently there is a new development of ice breaking super tankers for transporting liquefied natural gas (LNG) from the Yamal Peninsula to Asia and Europe (Gascard et al. 2017).

Model simulations under three different climate scenarios, performed during the ACCESS project, indicate that warmer climate close to the pole could trigger significant increase in tourism in Arctic countries between 2009 and 2085, in particular in Russia, Canada and Nunavut (Tol and Walsh 2015).

### V. What environmental impacts are Arctic economic activities likely to generate?

Expected changes of human activities—like an increase of ship traffic, partly due to redirection from southerly routes, and oil and gas exploitation activities in the Arctic—will impact local and regional air quality, and will have consequences for anthropogenic global warming (e.g., Granier et al. 2006; Corbett et al. 2010). The knowledge of the chemical behaviour of atmospheric pollutants is, however, sketchy.

Efforts to perform direct measurements of chemical compounds in the plumes of ships and fossil fuel extraction facilities under Arctic conditions were done as part of ACCESS (Law et al. 2017). These direct observations, to a large extent first time measurements, revealed deficiencies in existing inventories of emissions from these activities. For example, the intermittency of emissions due to flaring of surplus gas during oil extraction is underestimated and some chemical compounds from some of the sources are missing in the inventories. Also, for ship emissions, new estimates based on ACCESS observations revealed that NO_*x*_ emissions had been underestimated in cases when sea ice was present. The inventories are important sources of information when it comes to simulating current and future atmospheric pollution and the interaction of different chemicals in the atmosphere. Updated simulations highlighted that emissions from ships and from resource extraction facilities north of the Norwegian coastline significantly impact the composition and quality of the Arctic atmosphere already today (e.g., ozone, black carbon), and will likely increasingly do so in the future.

In ACCESS, model simulations were also used to estimate the global impact of local Arctic emissions. Difficulties arise for example from the fact that in some cases, like for sulphur, the reduction of pollutant emissions benefits local air quality but has a negative effect on global warming.

Arctic shipping leads to a net cooling due to the sulphur emissions, while petroleum extraction contributes to warming. For the coming decades, however, an increase of shipping in the Arctic, also due to re-routing from southerly routes, would lead to a warming contribution to the global temperatures, partly due to a reduction of sulphur emissions following new regulations (Law et al. 2017). If the ships are forced to drive at safety speed in the presence of sea ice, this will further decrease emissions on the Arctic routes, in particular during melting and freezing seasons. Many factors influence the actual emissions, like ship and fuel type, however, the ACCESS project showed that the major one is the occurrence, thickness and distribution of sea ice (Schröder et al. 2017).

Enhanced direct measurements and numerical assessments would allow better informed decisions and further insights on Arctic air pollution. Increased shipping activity and extraction of petroleum resources in Arctic waters would also increase the risk for oil spills. Thus important research topics in the ACCESS project included assessing the current oil-spill response capabilities (Wilkinson et al. 2017) and investigating how fate and footprint of an oil spill would change in a future climate, with changed seasonal sea-ice-cover. Numerical ensemble simulations were used to investigate six potential oil-spill scenarios, encompassing well blowouts, pipeline leaks and ship accidents at different locations (Nordam et al. 2017). Concerning oil spills, the increased length of the ice-free, or low ice-cover, season is a major difference between current and future climate in the Arctic because seasonal variation is larger than the change between the present and the situation projected until the middle of the century. Important factors influencing the impacts of an oil spill include the season when the spill occurs and its location. Simulations showed that sea ice had a huge impact on how the spilled oil was distributed over different environments, such as the sediment, the beaches, the water column or the atmosphere (evaporation). The many factors influencing the actual spill and its impact limit the potential to generalize results. For example, in coastal regions, sea ice can act as a shield to protect the coastline from the oil, or instead trap it, in case the oil spill occurs between the sea ice and the coast. However, project results pointed towards an overall increased risk due to oil spills. In addition to expected higher levels of activities, which could trigger an oil spill, the reduced sea-ice-cover may lead to a larger areal cover and more exposed shorelines. Oil-spill modelling in ice-covered waters is a developing field and further research is needed. The process is complicated not only due to sea-ice behaviour, but also the complex interactions between oil and sea ice.

The project also assessed current oil-spill response capabilities in terms of detection and monitoring, response techniques, and key scenarios (Wilkinson et al. 2017). Detection of oil in ice-covered waters (between, on, and in sea ice) poses new challenges to monitoring techniques and requires sophisticated sensors and underwater vehicles. Often the areas where the oil may gather, for example in leads (open water in the sea ice) or at the ice edge, are particularly rich in wildlife, creating specific risks for those areas. An important aspect is the threat and the burden an oil spill poses on local and indigenous people, as first responders or sufferers from a polluted living and food resource area.

In addition, shipping and resources extraction lead to changes in the underwater sound environment, which could influence marine organisms. In particular, model simulations performed during the project revealed that increased shipping could generate high sound levels leading to acute hearing problems and signal masking for animals in the vicinity of the ship. The communication and sonar range of animals could be considerably reduced for long periods of time. Hence ships should pass with sufficient distance from the protected areas and each other to avoid continuous masking (UPC 2014).

The marine spatial planning tool developed under ACCESS could be very useful in highlighting hotspots where pollution risks are higher and the environment particularly sensitive (Edwards and Evans 2017).

### VI. What are the expected impacts of climate change on indigenous peoples?

Arctic indigenous populations (about 400 000 individuals) live mostly around the Arctic Ocean in settlements ranging from modern cities to tiny villages.^10^ They share a long history of dealing with harsh conditions and environmental changes, despite their cultural diversity. The current rapid pace of climate change and its impacts raise concerns about adaptive capacity and sustainability. In addition to their impacts on economic activities and ecosystems, milder Arctic winters and retreating sea ice influence key aspects of Arctic indigenous peoples’ perceptions of vulnerability, resilience, risks and opportunities associated with climate change. These perceptions vary significantly with each culture’s livelihood and geographic location but a common trait is that climate change magnifies existing societal, political, economic, legal, institutional and environmental challenges.

Indigenous traditional livelihoods are economic choices based on reliance on local resources (hunting, herding, and gathering) but are also fundamental components of indigenous cultural identity. The higher nutritional value of traditional diets combined with the physical and spiritual benefits of outdoor harvesting activities bear great value for indigenous peoples’ health and spiritual wellness. However, climate change and environmental impacts strongly influence the health and availability of terrestrial and aquatic species harvested for food production. For example, ice retreat will likely threaten all ice-dependent seal species and risk to make seal harvests unsustainable. Hence the negative impacts of climate change on seal populations could also have direct consequences on the economic self-reliance, capacity building and cultural identity of some communities, in particular Inuits living in remote northern settlements.

Turning to or increasing activities in other economic sectors may become a necessity and a challenge, depending on the employment potential offered by other sectors and the possibility for indigenous hunters to integrate these sectors. Communities perceive invasive species moving into Arctic waters as a result of climate change simultaneously as new economic assets and threat. More extreme weather conditions like stronger winds also hinder food production activities such as fisheries. Ecosystem changes resulting from ocean acidification may also affect harvest and cultural practices. Atmospheric pollution from short-lived climate forcers such as black carbon (Law et al. 2017) represents an important threat for Arctic indigenous peoples’ health.

### VII. What constraints does a changing climate impose on Arctic governance and infrastructure?

Infrastructure includes governance frameworks (international agreements, regulations, soft law) as well as material-based infrastructure (ships, ports, communication networks, and observing networks for navigation and for monitoring the environment, pollution and climate change) (Dahms and National Research Council 1987; Niskanen 1991). Infrastructure development must be framed in the context of Arctic change driven by climate change, the evolution of the global demand for Arctic products strongly related to the global economy, pervading uncertainties (climatic, political and economic), and potential rapid change in a harsh and remote environment. Two important elements will likely control infrastructure development: (1) international harmonization of policies and regulatory regimes based infrastructure and (2) funding of material-based infrastructure which could involve private and public actors.

The ACCESS project aimed to identify the gaps in the existing governance regimes in the context of pan-Arctic governance and point out options for Arctic marine shipping, tourism, resource extraction, fishing and aquaculture in the light of potential climate change and pervasive uncertainties over a 30-year period (NERC 2015). Important gaps and limitations in the existing policy and regulatory framework for fisheries include the lack of coverage of high sea areas (outside of national legislated zones) by the current Regional Fisheries Management Organisation; the limited application of the UN Fisheries Stocks Agreement (only straddling and highly migratory fish stocks, are regulated, not shared and anadromous fish stocks); a general lack of fisheries related data to inform science-based governance decisions; and large heterogeneity and sometimes insufficiencies in coastal state regulations. Within the foreseeable future, most changes in fisheries regulation are likely to fall within the exclusive economic zones—hence within national rather than international regulation. Existing regulations dealing with port state controls and illegal, unreported and unregulated fishing may need to be enforced, and amended if necessary. Issues likely to require new regulations include vessels seeking new fishing opportunities in the central Arctic Ocean (NERC 2015). Recent negotiations between the five Arctic Ocean coastal states, Japan, Iceland, South Korea, China, and the EU seem to have made substantial progress after the end of the project toward a legally binding agreement to prevent unregulated fishing in the central Arctic Ocean.^11^

Aquaculture activities occur entirely in coastal waters and hence the implementation of governance regimes falls within individual states. The complex array of environmental and socio-economic changes facing northern communities requires an inclusive and integrated multi-stakeholder approach to aquaculture governance. Reviews of, for example, existing licensing, animal health, and construction of facilities regulations will be necessary in the light of climate change effects (NERC 2015).

ACCESS research illustrates that the main governance challenges facing marine transport, are the unification of the application and enforcement of ship rules. The new International Maritime Organization Polar Code fills many of the earlier gaps in shipping legislation in polar environments. However, it does not cover all polar marine safety and environmental protection issues and barely addresses the impacts of climate change. An International Convention for the Control and Management of Ships’ Ballast Water and Sediments will enter into force in September 2017. Further challenges are the inclusion of coastal communities (for example local economic and fishery interests); environmental protection and pollution prevention; international economic interests (Arctic natural resource developments), regional and local administration governance, and spatial planning. Significant gaps in regulation of Arctic shipping relate to insurance, liability and compensation in case of accidents. The current international system for compensation of pollution damage from ships is fragmented and limited. The geography of the Arctic Ocean as a closed sea makes transboundary pollution impacts one of the most difficult issues facing the legal and policy community (Rosen and Asfura-Heim 2013). Separate conventions address oil pollution liability and compensation from tankers; damage from the spill of bunker fuel carried in ships other than tankers, such as cargo ships; and hazardous and noxious substance spills from ships. None of the conventions address damage to the high seas beyond national jurisdiction (NERC 2015).

The current regulatory regime for oil and gas related activities varies between states and is fragmented. Coastal states implement, monitor and enforce regulations. No convention addresses liability and compensation arising from offshore oil rigs, pipelines and production systems (NERC 2015). Current public management and governance capacity in the Arctic is scattered across national and international authorities as well as global and local stakeholders, despite efforts to come to international regulations. For example, regulations relating to Arctic offshore oil and gas activities need to be strengthened and harmonized while taking into account differences in local conditions in terms of type of resource, infrastructure in place, local and indigenous communities.

Safe navigation in Arctic ice-covered waters part of the year, in particular along the NSR and the North West Passage (NWP), requires ports and infrastructure. Ongoing developments include super sites around the Yamal Peninsula (Sabetta) in Russia (the Yamal LNG project^12^) and Cambridge Bay in Canada (the Canadian High Arctic Research Station, CHARS^13^). The Yamal LNG project is mainly supported by private investments (15 000 million USD) for taking advantage of gas from the Yamal Peninsula. The Canada for the Cambridge Bay High Arctic Research Station (CHARS) is mainly supported by governmental investments Canada (250 million USD) for scientific research. There are also concerns from Greenland and Iceland for future Arctic marine infrastructure related to transpolar destinational shipping for exploiting Arctic mineral and live resources.

New developments after the end of the project include for example the formation by the Arctic Council of a Task Force on Arctic Marine Cooperation (April 2015) and an agreement on enhancing international Arctic scientific cooperation (11 May 2017). The ACCESS project with its 27 different partners from 9 European countries and the Russian Federation convened more than 80 researchers from a wide range of scientific disciplines and stakeholders. This kind of transdisciplinary Arctic scientific cooperation can provide the mix of knowledge overview and detail about Arctic development, which is needed to address the complex coupled challenges that the Arctic is already facing.

### VIII. What kind of management support would help understand and address the complex dynamics triggered by climate change?

Governance must find ways to grasp the most important impacts of a particular change, also in the geographical context where they occur. Marine spatial planning is a promising tool for this purpose and Norway has put substantial efforts to develop ecosystem-based management and marine spatial planning in the Arctic and in particular the Barents Sea (e.g., Olsen et al. 2007; Arctic Council 2013). Other Arctic governments have been slower to advance marine spatial planning and pan-Arctic initiatives are limited (Ehler 2014). Governance mechanisms and policy instruments must also be adaptive to respond in a proper way and within appropriate time scales. In addition, the large uncertainties associated with the non-negligible risks of tipping points motivate precautionary approaches including sometimes even safe standards (Margolis and Nævdal 2008; Crépin and Folke 2015).

ACCESS developed several tools to support decision making and management in the Arctic. These tools can help decision makers in general (like larger companies, regional governments, the Arctic Council) and policy makers in particular to better deal with the changes, because they also help to better understand principal characteristics of the system. The latter implies a tight link between science and policy, much tighter than exists today. The more change is expected the more important it is that decision makers understand the basic features of the most relevant processes in the system so that they can set in place appropriate response.

To that end ACCESS provided a marine spatial planning tool (Edwards and Evans 2017) and a framework for integrated ecosystem-based management (Crépin et al. 2017) that can be used jointly or separately. In contrast to most existing marine spatial plans that focus on particular Arctic regions, the marine spatial planning tool developed under ACCESS has a pan-Arctic scope. It is also a tool rather than a plan and provides a unique online interface that can be used and built on for all kinds of user-defined purposes (Edwards and Evans 2017). The framework for integrated ecosystem-based management goes beyond traditional ecosystem-based management and also incorporates economic and social dynamics. It provides decision support even in cases of scarce data and helps identify potential tipping points; it can also be easily built on as new tools, models, and scientific findings develop (Crépin et al. 2017). Its top down approach provides a good complement to the bottom up approach used in a resilience assessment (Arctic Council 2016) and can also be combined with it.

Quality and accessibility of data is also important for management support, the ACCESS project developed a climate data management system (Godøy and Saadatnejad 2017) and a set of indicators for sustainable development in the key economic sectors (Crépin et al. 2014; Petrick 2015; Schwarz et al. 2015). These complement the Arctic monitoring programme data and point to gaps with regard to the availability of socio-economic data, which often are only available at the national level although the Arctic Human Development Report (AHDR 2004) for example did make an effort to extract socio-economic data specific to the Arctic. In addition there is often a mismatch between temporal and geographic resolution between socio-economic and natural data, which makes good empirical studies of social–ecological interactions in the Arctic particularly challenging.

## Discussion and conclusion

The Arctic Ocean is a complex adaptive system in which different parts interact in an intricate and often unexpected manner. Geophysical, ecosystem and socio-economic dynamics in and outside the Arctic are tightly interlinked in complex ways. These interactions occur across spatial and temporal scales where global phenomena like climate change fundamentally alter living conditions for local and indigenous populations today and in the future, and Arctic resources such as stocks of marine seafood, oil, gas, and minerals raise global interests.

The EU funded research project ACCESS was one attempt to improve the knowledge about how these factors may interact over the next three decades. Natural sciences, social sciences and stakeholders worked jointly across disciplines and sectors to enhance our understanding of this coupled system. Here we provided an overview over results from the project, as far as they helped us answer questions of societal relevance, as to what changes in the Arctic Ocean we may anticipate, in the natural system and in the human use, and the feedbacks between them.

We can conclude that the combined natural and human system in the Arctic, despite all research efforts in the past, is subject to high levels of uncertainty in almost all fields. For the evolution of the physical natural system in the next decades, including the atmospheric regimes, air and ocean temperatures and sea ice, the largest uncertainties stem from natural variability that is inherent to the system. This uncertainty will pertain even more for ocean acidification, primary production, and higher trophic levels, including fish. Furthermore uncertainties regarding the economic development of the Arctic in the different marine sectors will interact with the economic and political situation in the rest of the world, not the least via the hugely influential oil and gas prices, which impact all economic activities in the Arctic Ocean. Science will likely not completely resolve these uncertainties due to the complex adaptive nature of the Arctic social–ecological system.

There are, however, things we do know and can take into account. The sea-ice thickness and summer sea-ice extent in the Arctic will continue to decrease; air and water mean temperatures tend to increase on a timescale of decades. Even if it is unsure when exactly most of the Arctic will become ice-free in summer, for practical purposes like shipping along the southern rim, a mostly sea-ice-free passage can be expected much earlier.^14^ However, despite longer ice-free seasons favouring shipping, model experiments suggest that blockages and a very mobile sea ice will still be a problem and safety threat. We do know that the extraction of additional fossil fuel from the Arctic will enhance the pressure on the global climate and contribute to trespassing the 2 °C, let alone the 1.5 °C warming limit goal according to the COP21 Paris Agreement. However Arctic gas extraction aimed at replacing coal could contribute towards achieving the Paris Agreement. We do know that interest in Arctic oil and gas, seafood, and transportation options is high and will likely stay high, following the potential rising demand elsewhere. This demand sets high stakes for management and governance in particular at international level, to minimize risks for the people and the environment. It is also clear that unless massive infrastructure investments are made, any activity in the Arctic will face issues of communication, safety and environmental risks.

Hence, appropriate governance must face those uncertainties and act upon available scientific information. While science cannot resolve all the uncertainties involved, management would benefit from scientific help to characterize the uncertainties involved and define the range of possible outcome to be aware of. Governance mechanisms and policy instruments must be adaptive to respond in a proper way and within appropriate timescales. Rapid changes imply the risk of either making policy out of date before it is even implemented or rushing through agreements based on the lowest common denominator, even when the highest standards would have been needed. Management support for the Arctic would have to address such kind of trade-offs. There is also a need to investigate possible consequences of alternative policy measures, for example whether to act upon available knowledge or postpone action to gather more information. The large uncertainties associated with the non-negligible risks of tipping points motivate precautionary approaches including sometimes even safe standards (see, e.g., Crépin and Folke 2015).

This ACCESS *Ambio* special issue is addressing major key challenges and issues related to Arctic climate change and development of human activities in the Arctic in order to provide some solutions and options from a marine perspective. Many challenges remain despite these extensive contributions. Here we list some of the more pressing ones:

- Regulations relating to Arctic offshore oil and gas activities must be strengthened and harmonized while taking into account differences in local conditions in terms of type of resource, infrastructure in place, and local and indigenous communities. The new Polar Code for shipping, the SAR agreement and the Fairbanks Agreement on enhancement of scientific cooperation are good examples but the details for their implementation still need to be specified. Similar regulations of oil spill response, Arctic tourist activities, and associated infrastructure, require prompt action.
- New key developments in physical infrastructure will certainly concern communication (broadband) in Polar Regions. No existing technology is available at the moment at the needed scale (pan-Arctic) but technical solutions exist, although expensive. Many challenges pertain for marine transportation like the lack of charts, training of polar operators and ice navigators, the development of an Arctic marine traffic awareness system, and the implementation of recent international agreements like the IMO Polar Code, the Arctic SAR and the Arctic Oil Pollution Preparedness and Response Agreements.
- Sound facts are a good basis for all governance decisions. Hence infrastructure for supporting scientific observations is a top priority. There is an urgent need to increase and improve observations in the Arctic atmosphere, ocean and sea-ice at a pan-Arctic scale and also at the regional scale. This is important not only to better understand processes but also a prerequisite to be able to parameterize and simulate them. Better observations are also necessary to improve weather forecasts, urgently needed for all kinds of activities in the Arctic, in particular for human and environmental safety reasons. The technology has improved to such extent that it is now conceivable to set up a proper Arctic observing network (SAON). This would involve observations from space (satellites) including some ground truth for validation and in situ components mainly composed of fixed (Eulerian) and mobile (Lagrangian) platforms for the ocean, the atmosphere and the cryosphere. ACCESS encourages coordination in the surveillance of marine ecosystems that are subject to climate variability and climate change beyond the Arctic proper, to include for example the Iceland fisheries. Experiences from the project INTERACT^15^ may be adapted to Arctic marine conditions. This system should be conceived in a way that includes the critical linkages between the Arctic, its actors and the rest of the world.
- There is a pressing need to address the lack of socio-economic data for the Arctic. Such data should be collected in ways and at spatiotemporal intervals such that it can be used jointly with biogeophysical data in a meaningful way. This would allow a better understanding of social–ecological and cross-sectoral interactions and improve forecasting capacity in all domains where human–nature interactions matter. Ideally a socio-economic data observing system should be part of the initiatives already discussed for biogeophysical data just mentioned (e.g., SAON). Other data needs concern quantification and understanding of the provision of ecosystem services and data with high enough resolution and number of observations to help anticipate and analyze potential abrupt changes and tipping points in all domains.
- Decision making based on state of the art scientific knowledge and advice requires more quantified and specific approaches to assess impacts. Governance tools better adapted to fulfil multiple goals could be developed building on tools like integrated ecosystem-based management, marine spatial planning, constructive and carefully chosen indicators, and resilience assessments (Arctic Council 2013, 2016).
- Any management action should also account for people’s potential reactions to such action because anticipation of some changes may trigger stronger reactions than the actual changes. People also often have general difficulties in interpreting risk and probabilities. In that context it may matter for example how potential future changes (e.g., in resource stock abundance, market conditions, policies and management strategies) are communicated. Visualization tools and coordination devices may help people take better informed decisions. (Lindahl et al. 2016, 2017).
- The policy-making process in the Arctic needs to actively incorporate traditional knowledge. National and industry interests should not systematically be allowed to override those of the environment or indigenous and local populations. We are convinced of the benefits of retaining a dialogue between non-Arctic States and the Arctic Council, in agreement with international law requirements for High Seas fisheries and Seabed areas beyond national jurisdiction (UNCLOS Art. 123). An active dialogue between all international stakeholders involved in Arctic governance issues is essential for successful and sustainable development and the wellbeing of the people. Standardization/harmonization of regulations would be ideal for all activities and in particular for transboundary live and mineral resources. For this to succeed there needs to be a commitment beyond the national level.

## Electronic supplementary material

Below is the link to the electronic supplementary material.

Supplementary material 1 (PDF 119 kb)

## Footnotes

1 Developing Arctic Modelling and Observing Capabilities for Long-term Environmental Studies, Framework Programme (FP) 6.

2 Arctic Tipping Points, FP7. See *Ambio* special issue: https://link.springer.com/article/10.1007/s13280-011-0230-9.

3 See project website: http://access-eu.org/en/publications.html.

4 The French combined Ice, Atmosphere, Arctic Ocean Observing System buoys (Gascard 2012).

5 More details and additional references on this particular topic are available in the electronic supplementary material to Crépin et al. (2017).

6 Carbon intensive activities instead of investing in clean production tend to move to countries with low carbon price.

7 See for example Ciccarelli (2014) “Warming up to Arctic Prospects” *Washington Post*, 5th of June 2014 or Struzik (2016) “Shipping Plans Grow as Arctic Ice Fades” *Yale Environment 360*, 17th of November 2016.

8 There are two routes namely the Northern Sea Route (NSR) along the Arctic Coast of Russia from Kara Gate to Bering Strait and the Northwest Passage (NWP) from Bering Strait through the Canadian Archipelago to Baffin Bay.

9 Based on United Nations Comtrade data which does not include Taiwan.

10 The information from this section was collected during an ACCESS workshop, from the ACCESS newsletter nr. 10 (http://access-eu.org/en/publications/access_newsletter.html), and an unpublished ‘ACCESS synthesis’ manuscript.

11 Meeting on high seas fisheries in the central Arctic Ocean: chairman’s statement: https://www.state.gov/e/oes/ocns/opa/rls/269126.htm retrieved 7 September 2017.

12
http://www.yamallng.ru/en/project/about/. Accessed 09-11-2017.

13
http://www.cbc.ca/news/canada/north/chars-arctic-research-station-climate-change-1.3484503. Accessed 09-11-2017.

14 During August 2017, a Russian-owned tanker, built for Arctic conditions completed a journey in record time from Europe to Asia, for the first time without ice-breaker (New York Times, 25 August 2017).

15 International Network for Terrestrial Research and Monitoring in the Arctic, European Union project, http://www.eu-interact.org/, retrieved 18 August 2017.
