# Supplementary material for: Arctic Climate Change, Economy and Society (ACCESS): Integrated perspectives
Source: Ambio. 2017 Oct 24;46(Suppl 3):341–54. doi: 10.1007/s13280-017-0953-3 (PMC5673869; doi:10.1007/s13280-017-0953-3)
Supplement: Supplementary file 1 — Supplementary material 1 (PDF 119 kb) [file 13280_2017_953_MOESM1_ESM.pdf]

Ambio

Electronic Supplementary Material

Title: Arctic Climate Change, Economy and Society (ACCESS): Integrated perspectives

Authors: Anne-Sophie Crépin, Michael Karcher, Jean-Claude Gascard

Publications of the ACCESS project by autumn 2017

Journal articles

Acosta Navarro, J-C., V. Varma, I. Riipinen, Ø. Seland, A. Kirkevåg, H. Struthers, T. Iversen, H.-C. Hansson & A. M. L. Ekman. 2016. Amplification of Arctic warming by past air pollution reductions in Europe. Nature Geoscience 9, 277-281. doi:10.1038/NGEO2673

Acosta Navarro, J-C. et al. 2016. Future response of temperature and precipitation to reduced aerosol emissions as compared with increased greenhouse gas concentrations. Journal of Climate, Volume 30(3). doi:10.1175/JCLI-D-16-0466.1

Bentsen, M., I. Bethke, J. B. Debernard, T. Iversen, A. Kirkevåg, Ø. Seland, H. Drange, C. Roelandt, I. A. Seierstad, C. Hoose, and J. E. Kristjánsson. 2014. The Norwegian Earth System Model, NorESM1-M. Part 1: Description and basic evaluation. Geosci. Model Dev., 6: 687-720. doi:10.5194/gmd-6-687-2013.

Berkman, Paul. Geopolitics of Arctic sea-ice minima. Brown J. World Aff. 19 (2012): 145.

Berkman, P.-A. 2012. Our Common future in the Arctic Ocean. The Round Table, Vol. 101, Issue 2: 123-135. doi:10.1080/00358533.2012.661527

Brigham, L. (2015). Future Perspective: The Maritime Arctic in 2050. *Fletcher F. World Aff.*, *39*, 109.

Brock, W. A., G. Engström, D. Grass , A. Xepapadeas. 2013. Energy balance climate models and general equilibrium optimal mitigation policies. Journal of Economic Dynamics and Control 37: 2371-2396. doi:10.1016/j.jedc.2013.09.008

Cassiani, M., Stohl, A., Olivié, D., Seland, Ø., Bethke, I., Pisso, I., and Iversen, T. 2016. The offline Lagrangian particle model FLEXPART-NorESM/CAM (v1): Model description and comparisons with the online NorESM transport scheme and with the reference FLEXPART model. Geosci. Model Dev., 9, 4029-4048. doi:10.5194/gmd-9-4029-2016

Crépin, A.S., Gren, Å, Engström, G. and D. Ospina. 2017. Operationalising a social-ecological systems perspective on the Arctic Ocean in Climate Change Economy and Society in the Arctic Ocean. Eds. Gascard, J.-C., A.-S. Crépin, M. Karcher and O.Young. eds. Special issue of *Ambio*.

Crépin, A.S., Karcher, M. and Gascard J.C. 2017. Arctic climate change, economy and society - integrated perspectives in: Climate Change Economy and Society in the Arctic Ocean. Eds. Gascard, J.-C., A.-S. Crépin, M. Karcher and O.Young. eds. Special issue of *Ambio*.

Dalsøren, S. B., Samset, B. H., Myhre, G., Corbett, J. J., Minjares, R., Lack, D., and Fuglestvedt, J. S. 2013. Environmental impacts of shipping in 2030 with a particular focus on the Arctic region. Atmos. Chem. Phys., 13, 1941-1955. doi:10.5194/acp-13-1941-2013

Dalsøren, S. B., Myhre, C. L., Myhre, G., Gomez-Pelaez, A. J., Søvde, O. A., Isaksen, I. S. A., Weiss, R. F., and Harth, C. M.: Atmospheric methane evolution the last 40 years, Atmos. Chem. Phys., 16, 3099-3126, doi:10.5194/acp-16-3099-2016, 2016.

Divine, D. V., Granskog, M. A., Hudson, S. R., Pedersen, C. A., Karlsen, T. I., Divina, S. A., Renner, A. H. H., and Gerland, S. 2015. Regional melt-pond fraction and albedo of thin Arctic first-year drift ice in late summer. The Cryosphere, 9, 255-268. doi:10.5194/tc-9-255-2015

Divine, D., C.-A. Pedersena, T.-I. Karlsena, H. Faste Aas, M.-A. Granskog, S.- R. Hudson, S. Gerland. 2016. Photogrammetric retrieval and analysis of small scale sea ice topography during summer melt. Cold Regions Science and Technology 129: 77-84. doi:10.1016/j.coldregions.2016.06.006

Dmitrenko, I. A., V. V. Ivanov, S. A. Kirillov, E. L. Vinogradova, S. Torres-Valdes, and D. Bauch. 2011. Properties of the Atlantic derived halocline waters over the Laptev Sea continental margin: Evidence from 2002 to 2009. J. Geophys. Res., 116, C1002. doi:10.1029/2011JC007269

Dmitrenko, I. A., S. A. Kirillov, V. V. Ivanov, B. Rudels, N. Serra, N. V. Koldunov. 2012. Modified Halocline Water over the Laptev Sea Continental Margin: Historical Data Analysis. Journal of Climate 25: 5556-5565. doi:10.1175/JCLI-D-11-00336.1

Dmitrenko I.A., S. A. Kirillov, N. Serra, N. V. Koldunov, V. V. Ivanov, U. Schauer, I. V. Polyakov, D. Barber, M. Janout, V. S. Lien, M. Makhotin, and Y. Aksenov. 2014. Heat loss

from the Atlantic water layer in the northern Kara Sea: causes and consequences. Ocean Sci., 10: 719-730. doi:10.5194/os-10-719-2014

Edwards, R. and A. Evans. 2017. The challenges of Marine Spatial Planning in the Arctic: Results from the ACCESS Programme in Climate Change Economy and Society in the Arctic Ocean. Eds. Gascard, J.-C., A.-S. Crépin, M. Karcher and O.Young. eds. Special issue of *Ambio*.

Eide, A. 2012. A bioeconomic MPA study based on cellular automata population growth and distribution. Fisheries Research, Vol.113, n°1: 118-132. doi:10.1016/j.fishres.2011.10.004

Eide, A. 2016. Management performance indicators based on year-class histories. Fisheries Research, 174:280-287. doi:10.1016/j.fishres.2015.10.026

Eide, A. 2016. Causes and consequences of fleet diversity in fisheries: The case of the Norwegian Barents Sea cod fishery. Elem Sci Anth. 2016;4:110. doi:http://doi.org/10.12952/journal.elementa.000110

Eide, A., K. Heen, C. Armstrong, O. Flaaten and A. Vasiliev. 2012. Challenges and Successes in the Management of a Shared Fish Stock – The Case of the Russian–Norwegian Barents Sea Cod Fishery. Acta Borealia, Volume 30, Issue 1: 1-20. doi:20/010.1080/08003831.2012.678723

Eide, A. 2017. Climate change, fisheries management and fishing aptitude affect spatial and temporal distributions in the Barents Sea cod fishery in Climate Change Economy and Society in the Arctic Ocean. Eds. Gascard, J.-C., A.-S. Crépin, M. Karcher and O.Young. eds. Special issue of *Ambio*.

Field, M., L. Beguery, L. Oziel and J. C. Gascard. 2015. Barents Sea monitoring with a SEA EXPLORER glider. OCEANS 2015 - Genova, Genoa, 2015, pp. 1-5. doi:10.1109/OCEANS-Genova.2015.7271540

Fors, A. S., Divine, D. V., Doulgeris, A. P., Renner, A. H. H., and Gerland, S. 2017. Signature of Arctic first-year ice melt pond fraction in X-band SAR imagery. The Cryosphere, 11, 755-771. doi:10.5194/tc-2016-125

Gascard et al. 2017a. Future sea ice conditions and weather forecasts in the Arctic. Implications for Arctic shipping, in: Gascard, J.-C., A.-S. Crépin, M. Karcher and O.Young. eds. Climate Change Economy and Society in the Arctic Ocean.Special issue of *Ambio*

Gascard et al 2017b infrastructure paper, in: Gascard, J.-C., A.-S. Crépin, M. Karcher and O.Young. eds. Climate Change Economy and Society in the Arctic Ocean. Special issue of *Ambio*

Glantz, P., A. E. Bourassa, A. Herber, T. Iversen, J. Karlsson, A. Kirkevåg, M. Maturilli, Ø. Seland et al. 2014. Remote sensing of aerosols in the Arctic for an evaluation of global climate model simulations. J. Geophys. Res. Atmos., 119: 8169-8188. doi:10.1002/2013JD021279

Godøy, O. and Saadatnejad. 2017. ACCESS climate data management in Climate Change Economy and Society in the Arctic Ocean. Eds. Gascard, J.-C., A.-S. Crépin, M. Karcher and O.Young. eds. Special issue of *Ambio*.

Golovin P.N., Ivanov V.V. 2015. "Density Stratification Effects on the Ice Lead Heat Balance and Perennial Ice Melting in the Central Arctic." Russ. Meteorol. Hydrol.: 40-46. doi:10.3103/S1068373915010070

Hermansen, Ø. & K. Heen. 2012. Norwegian salmonid farming and global warming: Socio-economic impact. Aquaculture Economics & Management, Vol. 16, n°3: 202-221. doi:10.1080/13657305.2012.704617

Hoog, S., Berger, J., Myland, J., Clauss, G.F., Testa, D. and Sprenger, F. 2012. The Mooring Bay Concept for LNG Loading in Harsh and Ice Conditions. Paper No. OMAE2012-83841, pp. 537-544; 8 pages doi:10.1115/OMAE2012-83841

Hudson, S. R., M. A. Granskog , A. Sundfjord , A. Randelhoff , A. H. H. Renner , D. V. Divine 2013. Energy budget of first-year Arctic sea ice in advanced stages of melt. Geophysical Research Letters, Vol. 40, Issue 11. doi:10.1002/grl.50517

Hwang, B., P. Elosegui, J. Wilkinson. 2015. Small-scale deformation of an Arctic sea ice floe detected by GPS Small-scale deformation of an Arctic sea ice floe detected by GPS and satellite imagery. Deep Sea Research II 120: 3-20. doi:10.1016/j.dsr2.2015.01.007

Isaksen, J. R., Ø. Hermansen, O. Flåten. 2015. Stubborn fuel tax concessions: The case of fisheries in Norway. Marine Policy 52: 85-92. doi:10.1016/j.marpol.2014.10.028

Ivanov V.V., Alexeev V.A., Repina I.A., Koldunov N.V., Smirnov A.V. 2012. Tracing Atlantic Water signature in the Arctic sea ice cover east of Svalbard. Advances in Meteorology, vol. 2012, Article ID 201818, 11 pages. doi:10.1155/2012/201818

Iversen, T., M. Bentsen, I. Bethke, J. B. Debernard, A. Kirkevåg,, Ø. Seland, H. Drange, J. E. Kristjansson, I. Medhaug, M. Sand, and I. A. Seierstad. 2013. The Norwegian Earth System Model, NorESM1-M - Part 2: Climate response and scenario projections. Geosci. Model Dev., 6: 389-415. doi:10.5194/gmd-6-389-2013

Jackson, K., Wilkinson, J., Maksym, T., Meldrum, D., Beckers, J., Haas, C., & Mackenzie, D. 2013. A novel and low-cost sea ice mass balance buoy. *Journal of Atmospheric and Oceanic Technology*, *30*(11), 2676-2688. doi.org/10.1175/JTECH-D-13-00058.1

Jiao, C., M. G. Flanner, Y. Balkanski, S. E. Bauer, N. Bellouin, T. Berntsen, H. Bian, K. Carslaw et al. 2013. An AeroCom assessment of black carbon in Arctic snow and sea ice. Atmos. Chem. Phys., 14: 2399-2417. doi:10.5194/acp-14-2399-2014

Kaminski, T., Kauker, F., Eicken, H., and Karcher, M. 2015. Exploring the utility of quantitative network design in evaluating Arctic sea ice thickness sampling strategies. The Cryosphere, 9: 1721-1733. doi:10.5194/tcd-9-1735-2015

Kauker, F., Kaminski, T., Ricker, R., Toudal-Pedersen, L., Dybkjaer, G., Melsheimer, C., Eastwood, S., Sumata, H., Karcher, M. & Gerdes, R. 2015. Seasonal sea ice predictions for the Arctic based on assimilation of remotely sensed observations. The Cryosphere Discuss, 9, 5521-5554.. doi:10.5194/tcd-9-5521-2015

Kirkevåg, A., T. Iversen, Ø. Seland, C. Hoose, J. E. Kristjánsson, H. Struthers, A. Ekman, S. Ghan et al. 2013. Aerosol-climate interactions in the Norwegian Earth System Model - NorESM1-M. Geosci. Model Dev., 6: 207-244. doi:10.5194/gmd-6-207-2013

Law, K. S., Roiger, A., Thomas, J. L., Marelle, L., Raut, J.-C., Dalsoren , S., Fuglestvedt, J., Tuccella, P., Weinzierl, B., Schlager, H. 2017. Local Arctic air pollution: sources and impacts in Climate Change Economy and Society in the Arctic Ocean. Eds. Gascard, J.-C., A.-S. Crépin, M. Karcher and O.Young. eds. Special issue of *Ambio*

Lindahl T., Bodin Ö. And Tengö M. 2015. Governing complex commons: the role of communication for experimental learning and coordinated management. Ecological Economics (111): 111-120. doi:10.1016/j.ecolecon.2015.01.011

Lindahl, T., Crépin, AS. & Schill, C. 2016. Potential Disasters can Turn the Tragedy into Success. Environ Resource Econ (2016) 65: 657. doi:10.1007/s10640-016-0043-1

Longrée M, Hoog S. Backbone for Escape, Evacuation and Rescue From Arctic Facilities: A Systematic Approach. 2014. ASME. International Conference on Offshore Mechanics

and Arctic Engineering, *Volume 10: Polar and Arctic Science and Technology* ():V010T07A005. doi:10.1115/OMAE2014-23085.

Longrée M, Hoog S., Menze M.: Engagement in the Arctic – the 'Modular Arctic Hub (MODARC)' facilitates the 'kick-off'. 2015. Proceedings of the ASME 2015 34st International Conference on Ocean, Offshore and Arctic Engineering. OMAE2015, May 31 - June 5, 2015, Saint John's, Newfoundland, Canada, OMAE2015-41225

Marelle, L., Thomas, J. L., Raut, J. C., Law, K. S., Jalkanen, J. P., Johansson, L., ... & Weinzierl, B. (2016). Air quality and radiative impacts of Arctic shipping emissions in the summertime in northern Norway: from the local to the regional scale. *Atmospheric Chemistry and Physics*, *16*(4), 2359-2379.

Makkonen, R., Seland, Ø., Kirkevåg, A., Iversen, T., and Kristjánsson, J. E. 2014. Evaluation of aerosol number concentrations in NorESM with improved nucleation parameterisation. Atmos. Chem. Phys. 14: 5127-5152. doi:10.5194/acp-14-5127-2014

Myhre, G., Samset, B. H., Schulz, M., Balkanski, Y., Bauer, S., Berntsen, T. K., Bian, H., Bellouin, N. et al. 2013. Radiative forcing of the direct aerosol effect from AeroCom Phase II simulations. Atmos. Chem. Phys., 13: 1853–1877. doi:10.5194/acp-13-1853-2013

Nordam, T., Dunnebier, D.A.E., Beegle-Krause, CJ., Reed, M. and D. Slagstad. 2017. Impacts of climate change and seasonal trends on the fate of Arctic oil spills in Climate

Change Economy and Society in the Arctic Ocean. Eds. Gascard, J.-C., A.-S. Crépin, M. Karcher and O.Young. eds. Special issue of *Ambio*

Noring, M., Hasselström, L., Håkansson,C., Soutukorva, Å. & Å. Gren. 2016. Valuation of oil spill risk reductions in the Arctic, Journal of Environmental Economics and Policy, 5:3, 298-317, DOI: 10.1080/21606544.2016.1155499

Oziel, L., Sirven, J., and Gascard, J.-C. 2016. The Barents Sea frontal zones and water masses variability (1980–2011) Ocean Sci.,12, 169-184. doi:10.5194/os-12-169-2016, 2016

Oziel, L., Neukermans, G., Ardyna, M., Lancelot, C., Tison, J. L., Wassmann, P., Sirven, J & Gascard, J. C. 2017. Role for Atlantic inflows and sea ice loss on shifting phytoplankton blooms in the Barents Sea. Journal of Geophysical Research: Oceans. doi:10.1002/2016JC012582, 2017

Petrick, S., Riemann-Campe, K., Hoog, S., Growitsch, C., Schwind, H., Gerdes, R. and Rehdanz, K. 2017. Climate Change, Future Arctic Sea Ice and the Potential for European Arctic Offshore Oil and Gas Production in Climate Change Economy and Society in the Arctic Ocean. Eds. Gascard, J.-C., A.-S. Crépin, M. Karcher and O.Young. eds. Special issue of *Ambio*.

Raut, J.-C., Marelle, L., Fast, J., Thomas, J. L., Weinzierl, B., Law, K. S., Berg, L., Roiger, L., Easter, R., Heimerl, K., Onishi, T., Delanoe, J. and Schlager, H. : Cross-polar transport and

scavenging of Siberian aerosols containing black carbon during the 2012 ACCESS summer campaign, Atmos. Chem. Phys. Disc., doi:10.5194/acp-2016-1023, 2017.

Roiger, A., J.-L. Thomas, H. Schlager, K. Law, J. Kim, A. Schäfler, B. Weinzierl, F. Dahlkötter et al. 2014. Quantifying emerging local anthropogenic emissions in the Arctic region: the ACCESS aircraft campaign experiment. *Bulletin of the American Meteorological Society* 96.3: 441-460. doi:https://doi.org/10.1175/BAMS-D-13-00169.1

Samset, G. Myhre, M. Schulz, Y. Balkanski, S. Bauer, T. K. Berntsen, H. Bian, N. Bellouin et al. 2013. Black carbon vertical profiles strongly affect its radiative forcing uncertainty. Atmos. Chem. Phys., 13: 2423-2434. doi:10.5194/acp-13-2423-2013

Samset, B. H., G. Myhre, A. Herber, Y. Kondo, S. Li, N. Moteki, M. Koike, N. Oshima et al. 2014. Modelled black carbon radiative forcing and atmospheric lifetime in AeroCom Phase II constrained by aircraft observations. Atmos. Chem. Phys., 14: 12465-12477. doi:10.5194/acp-14-12465-2014

Sand, M., T. K. Berntsen, J. E. Kay, J. F. Lamarque, Ø. Seland, and A. Kirkevåg. 2013. The Arctic response to remote and local forcing of black carbon. Atm. Chem. Phys., 13: 211-224. doi:10.5194/acp-13-211-2013

Sand M. , Iversen T., Bohlinger P. Kirkevåg A., Seierstad I., Seland Ø. and Sorteberg A. 2015. A Standardized Global Climate Model Study Showing Unique Properties for the Climate Response to Black Carbon Aerosols. Journal of Climate (28): 2512-2526. doi:10.1175/JCLI-D-14-00050.1

Schill, C., T. Lindahl, and A.-S. Crépin. 2015. Collective action and the risk of ecosystem regime shifts: insights from a laboratory experiment. Ecology and Society 20(1): 48. doi:10.5751/ES-07318-200148

Schröder, C., Reimer, N. & P. Jochmann. 2017. Environmental Impact of Exhaust emissions by Arctic Shipping in Climate Change Economy and Society in the Arctic Ocean. Eds. Gascard, J.-C., A.-S. Crépin, M. Karcher and O.Young. eds. Special issue of *Ambio*

Schwarz, J. P., B. Weinzierl, B. H. Samset, M. Dollner, K. Heimerl, M. Z. Markovic, A. E. Perring, and L. Ziemba (2017), Aircraft measurements of black carbon vertical profiles show upper tropospheric variability and stability, Geophys. Res. Lett., 44, 1132–1140, doi:10.1002/2016GL071241

Sumata, H., R. Kwok, R. Gerdes, F. Kauker, and M. Karcher. 2015. Uncertainty of Arctic summer ice drift assessed by high-resolution SAR data,,J. Geophys. Res. Oceans, 120, doi:10.1002/2015JC010810.

Sumata, H., T. Lavergne, F. Girard-Ardhuin, N. Kimura, M. A. Tschudi, F. Kauker, M. Karcher, and R. Gerdes. 2014. An intercomparison of Arctic ice drift products to deduce uncertainty estimates, J. Geophys. Res. Oceans, 119, 4887–4921, doi: 10.1002/2013JC009724.

Sutherland, P., and J.-C. Gascard. 2016.Airborne remote sensing of ocean wave directional wavenumber spectra in the marginal ice zone. Geophys. res. Lett., 43. doi:10.1002/2016GL067713

Taskjelle, T., Hudson, S. R., Granskog, M. A., & Hamre, B. 2017. Modelling radiative transfer through ponded first-year Arctic sea ice with a plane parallel model.The Cryosphere Discuss., doi:10.5194/tc-2017-36, 2017, Manuscript under review for journal The Cryosphere,Discussion started: 13 March 2017

Thomas, J.L., J.-C. Raut, K. S. Law, L. Marelle, G. Ancellet, F. Ravetta, J. D. Fast, G. Pfister, L. K. Emmons, G. S. Diskin, A. Weinheimer, A. Roiger and H. Schlager. 2013. Pollution and transport towards Greenland during summer 2008. Atmos. Chem. Phys., 13, 3825-3848. doi:10.5194/acp-13-3825-2013, 2013

Troell, M., A. Eide, J. Isaksen, Ø. Hermansen and A.-S. Crépin 2017. Sea Food from a changing Arctic in Climate Change Economy and Society in the Arctic Ocean. Eds. Gascard, J.-C., A.-S. Crépin, M. Karcher and O.Young. eds. Special issue of *Ambio*

Tuccella P, Thomas JL, Law KS, Raut J-C, Marelle L, Roiger A, et al.. Air pollution impacts due to petroleum extraction in the Norwegian Sea during the ACCESS aircraft campaign. Elem Sci Anth. 2017; 5:25. DOI: http://doi.org/10.1525/elementa.124

Tsigaridis, K., Daskalakis, N., Kanakidou, M., Adams, P. J., Artaxo, P., Bahadur, R., Balkanski, Y., Bauer, S. E. et al. 2014. The AeroCom evaluation and intercomparison of

organic aerosol in global models. Atmos. Chem. Phys., 14: 10845-10895. doi:10.5194/acp-14-10845-2014

UPC (Universitat Politècnica de Catalunya) 2014. Simulator of the effects of noise from oil industry operations on marine mammals. ACCESS deliverable D4.52 (http://www.access-eu.org/en/deliverables2/wp4.html)

Wang, C., M. A. Granskog, S. Gerland, S. R. Hudson, D. K. Perovich, M. Nicolaus, T. I. Karlsen, K. Fossan, and M. Bratrein 2014. Autonomous observations of solar energy partitioning in first-year sea ice in the Arctic Basin. J. Geophys. Res. Oceans, 119, 2066–2080. doi:10.1002/2013JC009459

Wang, C., M.-A. Granskog, S.-R. Hudson, S. Gerland, A.-K. Pavlov, D.-K. Perovich, M. Nicolaus. 2016. Atmospheric conditions in the central Arctic Ocean through the melt seasons of 2012 and 2013: Impact on surface conditions and solar energy deposition into the ice-ocean system. Journal of Geophysical Research Atmospheres. doi:10.1002/2015JD023712

Wilkinson, J. P., Boyd, T., Hagen, B., Maksym, T., Pegau, S., Roman, C., ... & Zabilansky, L. (2015). Detection and quantification of oil under sea ice: The view from below. *Cold Regions Science and Technology*, *109*, 9-17. doi.org/10.1016/j.coldregions.2014.08.004

Wilkinson, J., Beegle-Krause, C.J., Evers, K.-U., Hughes, N., Lewis, A., Reed, M., and P. Wadhams. 2017. Oil spill response capabilities and technologies for ice-covered water: A review of recent developments and established practices in Climate Change Economy

and Society in the Arctic Ocean. Eds. Gascard, J.-C., A.-S. Crépin, M. Karcher and O.Young. eds. Special issue of *Ambio*

Book chapters

Berkman, P.A. 2012. Common interests' as an Evolving Body of International law: Applications for Arctic Ocean Stewardship. Arctic Science, International Law and Climate Change. ed. S. Wasum-Rainer, I. Winkelmann, K. Tiroch, 155-174. Heidelberg: Springer Berlin. doi:10.1007/978-3-642-24203-8_17

Berkman, P. A., Vylegzhanin, A. N. 2013. Preface: International, Interdisciplinary and Inclusive Perspectives. Environmental security in the Arctic Ocean. ed. P. A. Berkman, A. N. Vylegzhanin, 19-41. Springer Netherlands. doi:10.1007/978-94-007-4713-5

Berkman, P. A., Vylegzhanin, A. N. 2013. Chapter 32: Conclusions: Building Common Interests in the Arctic Ocean. Environmental security in the Arctic Ocean. ed. P. A. Berkman, A. N. Vylegzhanin, 371-404. Springer Netherlands. doi:10.1007/978-94-007-4713-5

Brock, W., G. Engström, and A. Xepapadeas 2015. Energy Balance Climate Models, Damage Reservoirs and the Time Profile of Climate Change Policy. The Oxford Handbook of the Macroeconomics of Global Warming. ed. L. Bernard and W. Semmler, 19-52. Oxford.

Eide, A. 2014. Modelling Spatial Distribution of the Barents Sea Cod Fishery. Cellular Automata. ACRI 2014. Lecture Notes in Computer Science, vol 8751. ed. Wąs J., Sirakoulis G.C., Bandini S., 288-299. Cham: Springer. doi:10.1007/978-3-319-11520-7_30

Gascard, J.-C. 2012. From the DAMOCLES to ACCESS Projects (Sixth & Seventh EU Framework Programmes 2005-2015) IAOOS – An Advanced Arctic Ocean Observing System (2011-2019). Arctic Science, International Law and Climate Change. "ed. S. Wasum-Rainer, I. Winkelmann, K. Tiroch, 261-283. Heidelberg: Springer Berlin. doi:10.1007/978-3-642-24203-8_1

Stammler-Gossmann, A. 2011. "Indigenous peoples": Roots of Russian minority politics („Indigene Völker": Wurzeln der russländischen Minderheitenpolitik). The Arctic Logbook. Environment, Economics, Politics. ed. Sapper, M, Weichsel, V., Humrich, Ch., 417-427. Berlin: Osteuropa.

Reports

Crépin, A.S. S. Petrick, E. Morgenroth, M. André, A. Eide, Ø. Hermansen, J. Isaksen, T. Lindahl, A. Stammler-Gossman and M. Troell. 2014. Indicators for sustainable seafood production. Beijer Discussion paper 249. http://www.beijer.kva.se/PDF/5175809_Disc249.pdf

Hermansen, Oystein, and Max Troell. "Aquaculture in the Arctic–a review." Marine Ecology Progress Series 373 (2012): 265-273.

Hoegh-Guldberg, O., R. Cai, E.S. Poloczanska, P.G. Brewer, S. Sundby, K. Hilmi, V.J. Fabry, S. Jung, W. Skirving , D.A. Stone, M.T. Burrows, J. Bell, L. Cao, S. Donner, C.M. Eakin, A. Eide, et al.(2014). The Ocean. in: IPCC. Climate Change 2014: Impacts, Adaptation, and Vulnerability. Part B: Regional Aspects; Contribution of Working Group II to the Fifth Assessment Report of the Intergovernmental Panel on Climate Change; Cambridge University Press: Cambridge, UK, 2014; p. 688.

Lindahl, T., A.-S. Crépin, and C. Schill. 2012. Managing resources with potential regime shifts: using experiments to explore social-ecological linkages in common resource system. Beijer Discussion Paper Series 232. Beijer Institute of Ecological Economics, Stockholm, Sweden.

Lindahl, T., A.-S. Crépin, and C. Schill. 2014. Potential disasters can turn the tragedy into success. Beijer Discussion Paper Series 244. Beijer Institute of Ecological Economics, Stockholm, Sweden.

Murray, M.S., Eicken, H., Starkweather, S., Gerlach, S.C., Evengaad, B., Gearheard, S., Schlosser, P., Karcher, M.P., et al. 2012. Responding to Arctic environmental change: Translating our growing understanding into a research agenda for action. Report on the 1st ISAC Responding to Change Workshop, 30 January – 1 February 2012, Kingston, Ontario. Stockholm/ Fairbanks: International Study of Arctic Change.

Schill, C. (2017). *Human Behaviour in Social-Ecological Systems: Insights from economic experiments and agent-based modelling.* Doctoral dissertation, Stockholm Resilience Centre, Stockholm University.

Stammler-Gossmann, A. 2011. Re-bordering of the Russian North. Arctic & Antarctic International Journal of Circumpolar Sociocultural Issues 5: 97-114.

Deliverables

A list of all ACCESS deliverables and links to the full test of those publically available is available online http://www.access-eu.org/en/deliverables2.html, retrieved August 29, 2017.
